# Evaluation of Electronic and Paper-Pen Data Capturing Tools for Data Quality in a Public Health Survey in a Health and Demographic Surveillance Site, Ethiopia: Randomized Controlled Crossover Health Care Information Technology Evaluation

**DOI:** 10.2196/10995

**Published:** 2019-02-11

**Authors:** Atinkut Alamirrew Zeleke, Abebaw Gebeyehu Worku, Adina Demissie, Fabian Otto-Sobotka, Marc Wilken, Myriam Lipprandt, Binyam Tilahun, Rainer Röhrig

**Affiliations:** 1 Division of Medical Informatics Department of Health Services Research Carl von Ossietzky University of Oldenburg Oldenburg Germany; 2 Department of Health Informatics Institute of Public Health University of Gondar Gondar Ethiopia; 3 Department of Reproductive Health Institute of Public Health University of Gondar Gondar Ethiopia; 4 Division of Epidemiology and Biometry Department of Health Services Research Carl von Ossietzky University of Oldenburg Oldenburg Germany

**Keywords:** public health, maternal health, surveillance, survey, data collection, data quality, tablet computer, mHealth, Ethiopia

## Abstract

**Background:**

Periodic demographic health surveillance and surveys are the main sources of health information in developing countries. Conducting a survey requires extensive use of paper-pen and manual work and lengthy processes to generate the required information. Despite the rise of popularity in using electronic data collection systems to alleviate the problems, sufficient evidence is not available to support the use of electronic data capture (EDC) tools in interviewer-administered data collection processes.

**Objective:**

This study aimed to compare data quality parameters in the data collected using mobile electronic and standard paper-based data capture tools in one of the health and demographic surveillance sites in northwest Ethiopia.

**Methods:**

A randomized controlled crossover health care information technology evaluation was conducted from May 10, 2016, to June 3, 2016, in a demographic and surveillance site. A total of 12 interviewers, as 2 individuals (one of them with a tablet computer and the other with a paper-based questionnaire) in 6 groups were assigned in the 6 towns of the surveillance premises. Data collectors switched the data collection method based on computer-generated random order. Data were cleaned using a MySQL program and transferred to SPSS (IBM SPSS Statistics for Windows, Version 24.0) and R statistical software (R version 3.4.3, the R Foundation for Statistical Computing Platform) for analysis. Descriptive and mixed ordinal logistic analyses were employed. The qualitative interview audio record from the system users was transcribed, coded, categorized, and linked to the International Organization for Standardization 9241-part 10 dialogue principles for system usability. The usability of this open data kit–based system was assessed using quantitative System Usability Scale (SUS) and matching of qualitative data with the isometric dialogue principles.

**Results:**

From the submitted 1246 complete records of questionnaires in each tool, 41.89% (522/1246) of the paper and pen data capture (PPDC) and 30.89% (385/1246) of the EDC tool questionnaires had one or more types of data quality errors. The overall error rates were 1.67% and 0.60% for PPDC and EDC, respectively. The chances of more errors on the PPDC tool were multiplied by 1.015 for each additional question in the interview compared with EDC. The SUS score of the data collectors was 85.6. In the qualitative data response mapping, EDC had more positive suitability of task responses with few error tolerance characteristics.

**Conclusions:**

EDC possessed significantly better data quality and efficiency compared with PPDC, explained with fewer errors, instant data submission, and easy handling. The EDC proved to be a usable data collection tool in the rural study setting. Implementation organization needs to consider consistent power source, decent internet connection, standby technical support, and security assurance for the mobile device users for planning full-fledged implementation and integration of the system in the surveillance site**.**

## Introduction

### Scientific Background

In most of the low- and middle-income countries, millions of people born and die without registration in any legal and statistical records. Health and social policy planning and evaluations are performed with statistical assumptions for unrecorded lives [1,2]. The lack of a fully functional civil registration system in those countries force them to rely on other interim measure data sources such as censuses, Health and Demographic Surveillance Systems (HDSSs), and Demographic and Health Surveys (DHS) programs to understand population- level health determinants [3,4]. Conducting any survey in those countries requires extensive use of paper-pen and manual processes to manage the data [5]. Paper data collection processes are labor intensive, time-consuming, susceptible to errors, incur high printing and running costs, and are cumbersome and uncomfortable for field data collection [6,7]. The need to support the paper process and the recent advanced popularity of mobile devices fortified the development and use of electronic data collection methods in community health and clinical research works. Electronics devices such as personal digital assistants [8-10], mobile phones [11,12], and tablet computers are noticeably tested for their potential role in replacing the standard paper-based tools [7]. The anticipation is that electronic data capturing tools may overcome substantial limitations of the paper-based system through saving time, improving the data quality, and minimizing overall cost [6,7,9,13,14]. Due the fast evolution of information technology and the heterogenicity of infrastructures in different countries, evaluations of such systems require periodic and setting context evidence to support the growing claims on their efficiency, effectiveness, and impacts [15,16].

### Rationale for the Study

The recent emerging research outputs are expediting the use of electronic data collection methods in lower- and middle-income countries. However, most of the available evidence cannot surpass the common critics on the quality of mobile health (mHealth) evidence. The critic shares 2 major points: First, there is little to no field-based study for quantifying the interaction of data quality and data capture technologies. Second, rigorous scientific research designs such as randomized controlled trials are few in number and type [17-20]. The majority of the research papers are work experience reports and simple descriptive one-arm studies [21-23]. Moreover, the comparative studies are from research conducted at a different time [7,9,11] or surveys that are not conducted on field and rather conducted in the hospital and clinic settings [10,12,24]. The presence of an interviewer adds an additional breadth to the multifaceted interaction among the survey questionnaire, the respondent, and the survey delivery approach, which can result in different approach effects. Therefore, use of smart devices such as a tablet computer to support interviewer-based survey questionnaires would be explored separately. There are experiences of using mHealth for data collection in Ethiopia [7,25,26]. According to the authors’ literature review and expertise, there is a lack of comparative trial evaluation studies on the effect of data capture tools on the quality of the data in interviewer-administered surveys conducted in demographic and surveillance sites.

Therefore, in this study, we evaluated mobile electronic data capture (EDC) tools and compared them with the traditional standard paper-based system on the quality of recorded responses from rural community household respondents.

### Objectives

The purpose of this study was to compare data quality parameters in the data collected using mobile electronic and standard paper-based data capture tools from Dabat Health and demographic surveillance sites in northwest Ethiopia.

The study’s primary research question was:

- Is the error rate of data collected by an EDC tool less compared with paper and pen recorded data?

The secondary questions were:

- Is there a learning effect in the use of EDC through the course of the data collection period?
- Is an electronic mobile-based data collection tool usable to the data collectors in Dabat health and demographic surveillance sites?

### Study Context

#### Organizational Setting

This evaluation research was implemented at Dabat HDSS, also called the Dabat Research Center, in northwest Ethiopia. The surveillance site is a member of the INDEPTH global network of HDSSs, which has 42 health research centers as members and 47 HDSS field sites in 18 low- and middle-income countries in Africa, Asia, and Oceania [27]. The surveillance center established in 1996 aims to generate community-based representative evidence through a continuous longitudinal data collection process. The surveillance is run by the College of Medicine and Health Sciences, which is one of the colleges/faculties of the University of Gondar in Ethiopia. Dabat district, the surveillance site, is one of the 21 districts in North Gondar Administrative Zone of Amhara Region in Ethiopia. According to the INDEPTH Network, Dabat HDSS is working with 69,468 participants [28].

### System Details and System in Use

A 30-page paper questionnaire, in 8 subthematic sections, was developed by a team of researchers at the Institute of Public Health at the University of Gondar, Ethiopia. The comprehensive survey was called “integrated survey on maternal, childhood, nutrition, and disability in Dabat Health and Demographic Surveillance System (HDSS).” The themes addressed pregnant women, young females, children aged less than 5 years, and people with disabilities. The number of questions in each subthematic questionnaire varies from 15 to 63, and each interviewer can use at least one of them or a combination of subthematic questionnaires based on the respondent’s category of cases. Most of the overall items (88.25%) were closed-ended questions.

#### Open Data Kit Questionnaire Development

**Figure 1:**
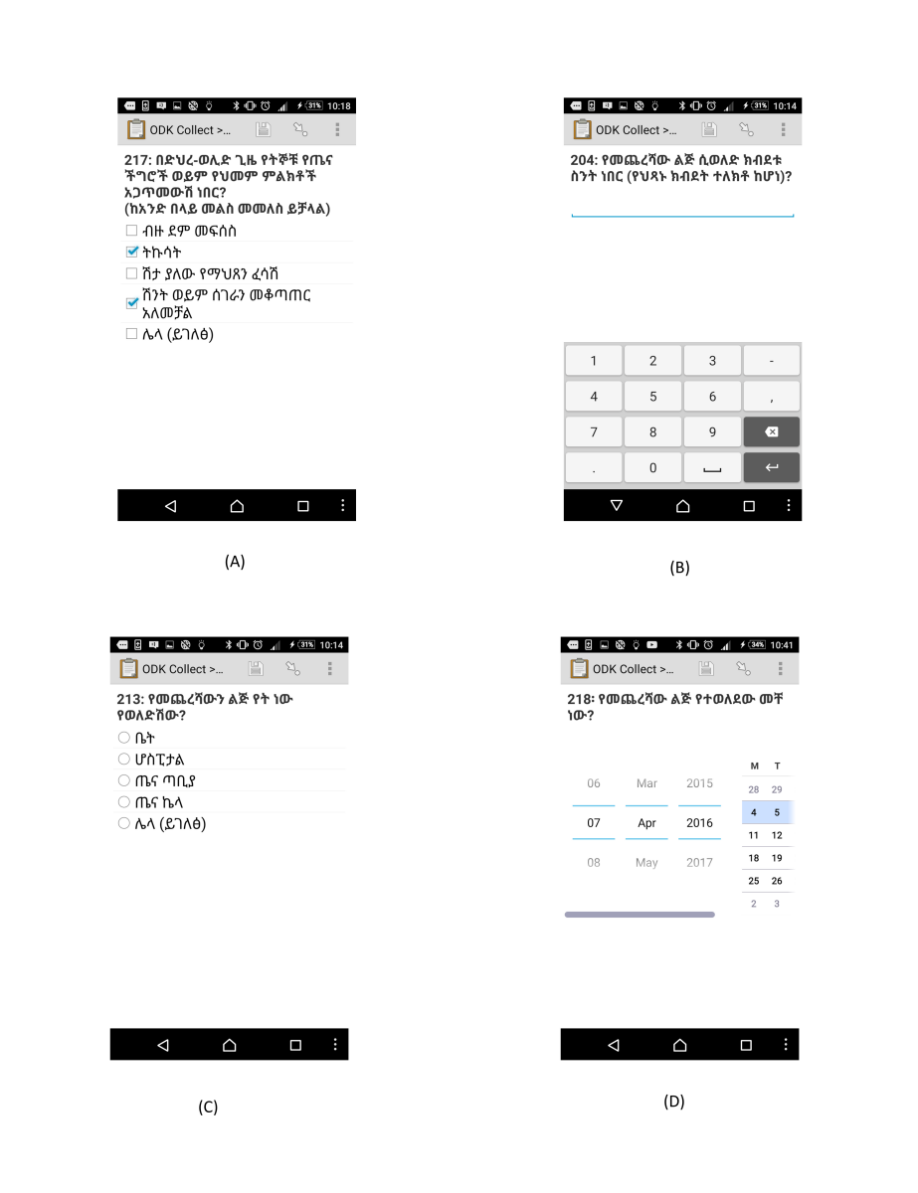
Screenshot examples of the type of questions; multiple choices (A), number (B), single select (C), and date (D) presented in the electronic data capture tool used for the survey in Dabat Demographic and Health Surveillance site in June 2016, northwest Ethiopia. Translation of the Screen: (A) 217: Which of the signs of pregnancy complications or danger sign of pregnancy have you encountered during postnatal period? bleeding, fever, vaginal gush of fluid, incontinency, and other (describe); (B) 204: Birth weight for the last child (if it was measured); (C) 213: Where did you give birth for your last child? home, hospital, health center, health post, and other (please mention it here); and (D) 218: When was the last child delivered?

Open Data Kit (ODK) was chosen to develop the EDC system using an open-source app. ODK has an open suite of tools used to design the form, collect, and aggregate the data. Similar experience was observed in a couple of recent studies [7,29]. The exact copy of the paper questionnaire was converted to its electronic replica form using Excel and XLSForm. After passing the technical validity, the form was uploaded to the server. Each data collector can get a new blank form for the first time with an authorized log-in to the server. For multiple-choice questions, single question per screen is displayed on the tablet screen, whereas for questions in a tabular form, many questions per screen are shown on the screen. From overall questionnaire items, only the respondents’ household identification (ID), personal ID, and data collectors’ and their supervisor’s name were required. The questionnaire is designed to filter numbers, assist date function, and check for skip patterns to prevent significant typing and data format errors. No other error controlling functionalities were incorporated because of the 2 data collectors per interview design nature of this study. The data entry fields were restricted to option buttons, check boxes, or empty fields (Figure 1).

After completing each interview, the data collector can use the “Edit Saved Formˮ function for any valid correction and can use the “Send Finalized Formˮ function to send the form to the server instantaneously. The third generation mobile internet network was used to connect each tablet computer to the server. In the case of limited network connectivity, submissions of the saved data were transferred when the data collectors were in a good network coverage area.

For this survey, we used the *TechnoPhantem7* tablet computer. The device is locally manufactured in Ethiopia with an inbuilt Ethiopian national working language called Amharic. A fully charged tablet has a battery life of approximately 48 hours. We have restricted the functionalities of many unnecessary apps in the devices to save the battery lifetime. To test the natural course of electricity infrastructure in the areas, we have not used extra battery or power banks as a reserve power source to charge the tablets.

## Methods

### Study Design

We conducted a field-based, prospective, randomized, controlled, crossover trial to investigate the error rate as an indicator of data quality. Moreover, usability evaluation using System Usability Scale (SUS) and semistructured questionnaire (Multimedia Appendix 1) interviews were conducted to observe the user impression in use of EDC.

The evaluation study started after the approval by the Ethical Committee of the University of Oldenburg (vote-number 148/2016, chair Professor Griesinger) and the ethical vote of the University of Gondar, Research and Community Service Gondar, Ethiopia (vote-number O/V/P/RCS/05/501/2015, signed by TT Adefris). The data are kept confidentially and the electronic copy of the data is stored in the Department of Medical Informatics of the University of Oldenburg

### Study Participants

Oral consent was obtained from the participants and data collectors after the objective of the research was explained briefly. Data collectors, who were permanently employed to Dabat DHS site and contractually employed for this integrated survey, were our research participants. The data collectors’ main task was to travel house to house and conduct a face-to-face interview with the persons living in selected Kebeles (smallest administrative unit) of Dabat district.

From 13 towns chosen for the overall survey, 6 towns were selected for our specific research based on accessibility of internet coverage and electric power supply in the town or nearby towns. On the basis of this, 12 data collectors, who were assigned to work in 6 towns, were involved. On each survey day, the interviewer was randomly selected to have either a tablet computer or a paper questionnaire based on computer- generated orders of tool users in the study period.

### Study Flow

The evaluation trial started after the technical team at the University of Gondar, Department of Health Informatics in Ethiopia managed the form creation, ODK Collect app installation on the tablet, and server configuration. Following this, we trained data collectors for 2 days on the basic tablet computer use, EDC functionalities, and questionnaire content. After incorporating comments from the participants on the functionality of the system, we performed pretests of the system for 2 days in towns outside from our trial area. The pretest phase showed some critical deficits of the system, and we have discovered unseen system errors, such as unnecessarily required items that significantly delay the data collection time. They were removed, and many decimal point items were corrected. A mini user manual using the local language was prepared and given to the interviewer along with working procedures.

The 2-interviewer pair, one with tablet computer and the other with paper questionnaire, went together to each household and sat next to each other to conduct the interviews (Multimedia Appendix 2). In a given interview, one of the data collectors leads and asks the questions, whereas both fill in the data independently and concurrently. For each interview day, the schedule of field data collectors were generated according to a computer-generated random order. The order defined the date and the data collection tool—EDC or paper and pen data capture (PPDC)—for each data collector in a team. The interviewers did not know the generated order in advance. Upon the supervisor notification, the paper and tablet devices were exchanged between the interviewers.

To evaluate the electronic tool, we used the first month of the comprehensive survey meant to address a larger population for approximately 4 months. Our actual data collection period was from May 10, 2016, to June 3, 2016. At the end of the field-based data collection period, the usability questionnaire was given to each data collector. Moreover, their satisfaction and opinion during the system use were asked by using a semistructured interview questionnaire (Multimedia Appendix 3).

### Outcome Measures or Evaluation Criteria

We chose the overall data quality errors (missing and inaccuracy) as indicators of data quality. It indicates the existing, missing, or inaccuracy errors from the data collected using either paper or electronic tools. The following are the evaluation criteria:

- Overall errors: This indicates the existence of one and more error types from the total records in a questionnaire (how many of the records have one or more items with errors from the total records). For the pen and paper questionnaire, we use the original responses recorded by the data collectors before correction is given from the supervisor or data passes to cleaning and data entry phases.
- Missing: It represents missing answers or questions with no answers in questionnaire completed by the interviewer and submitted to the supervisor or to the server.
- Inaccuracy: It represents any problematic items or incompatible values in the data. It includes decimal point errors, invalid date, or text-unreadable values.
- Quantitative SUS evaluation: Response from the 10-item questionnaire based on a 5-point Likert scale was summarized. The system was considered usable if the overall SUS score was >67 (ranging from 0-100) [30].
- Qualitative isometric: The analysis is based on the International Organization for Standardization (ISO) 9241-110 dialogue principle. The 7 dialogues were based on the description in the studies [31,32]. Transcribed interview response was related to the 6 dialogue principles and summarized with a “+” and “−” notation if the interview response fits positively or negatively, respectively.

### Methods for Data Acquisition and Measurement

The data collectors with paper questionnaires hand over completed questionnaires for their respective supervisors daily. To identify the potential errors in a given paper-based questionnaire, the supervisors used error extraction sheets and recorded all identified errors before giving correction, comments, and suggestions back to the data collectors. For the EDC system, the completed questionnaires were directly submitted to the server. The administrator has privileges to access and monitors the process daily and checks submissions from all interviewers. Technical inquiries from the data collectors or supervisors are replayed immediately by standby technical members.

Electronically submitted data were downloaded on a weekly basis in Excel format from the server and sent to an actual research site where the counter paper-based questionnaire remained. Furthermore, 2 technical assistants compared the data errors recorded on the extraction sheets and electronically submitted data. Each item in the questionnaire was double checked and the errors were noted accordingly.

Anonymized data from the study site were brought to a department of the Medical Informatics at the Carl von Ossietzky University of Oldenburg. MySQL was used for the cleaning and preparation of data for analysis by 2 researchers.

To evaluate the software usability, we used the Amharic language translated SUS questionnaire [30]. The 10-item questionnaire is based on a 5-point Likert scale and produces a maximum score of 100 on the users’ impression on the EDC tool. We chose SUS because of its shortness and the understandable phrasing of questions even for noninformation technology skilled persons such as our data collectors. The results of our SUS questionnaire showed a Cronbach alpha of .841 for reliability.

We also used semistructured questionnaires to perform face-to-face interviews with data collectors who were involved in this trial. Tape-recorded and noted data were then transcribed for further analysis based on the dialogue principles defined in the ISO 9241-110 [31].

### Methods for Data Analysis

Univariate analyses, to quantify errors in data capture tools, was performed by SPSS (IBM SPSS Statistics for Windows, Version 24.0) and R statistical software (R version 3.4.3, The R Foundation for Statistical Computing Platform) was used to perform ordinal logistics mixed regression model to see the effect of some variables on the quality of the data. As the dataset contains 2 values for each observation, a number of errors for the tablet questionnaire and a number of errors for the paper version, there are a lot of ties or dependencies between the observations. To reduce these ties, we constructed a new variable as the difference between the 2 numbers of errors. However, this variable contains many zeros. We chose to resolve this problem by categorizing the variable into the following 3 categories: fewer errors in the paper version than in the tablet version, zero difference, and more errors in the paper version than on the tablet. We assumed a dependence between the error rates and the overall length of the questionnaire as well as differences in performance between groups of the 6 interviewers. Consequently, we constructed a mixed ordinal regression model to explain the category of error differences along the length of the questionnaire. The model contains random effects for the individual performance of groups of interviewers. At the same time, we estimated models that specialized on the influence of the different types of questions and their quantities. For every model, we used a regression equation with the following structure:

Error_diff_cat = b_1 covariate + a_G1 covariate + … + a_G6 + e

1

“Covariate” stands for the different measures of the length of the questionnaire and a_G1 to a_G6 denote the random effects for each group. For the estimation, we used a cumulative logistic mixed regression model. Estimation was performed within the software R using the add-on package “ordinal.” We tried to include additional covariates in a forward selection process though they were eliminated on the basis of the Akaike Information Criterion. For each regression coefficient, we can construct a test for statistical significance of the influence of the corresponding covariate. We may test the hypotheses H0: |β|=0 versus H1: |β|≠0 by constructing a confidence interval for the level 1-α and check whether it includes the value from H0. Hence, we can conclude whether we find a significant increase in the error rate in either of the 2 methods.

For qualitative analysis, 2 computer scientist experts and 1 public health expert with a professional informatics background independently mapped the qualitative responses to 6 of the 7 dialogue principles, and then later approved the category together. When a difference arose, a physician and medical informatics expert was consulted for an agreement.

The dialogue principles of ISO 9241-110, namely, suitability for the task, suitability of learning, controllability, self- descriptiveness, conformity with user expectation (CUE), and error tolerance (ET) is used to map the transcribed interview result. To include many of the respondent views, we have expanded the dialogue principles, mainly suitability for the tasks, focused from the software and hardware context to include social and environmental contexts.

## Results

### Data Collectors’ Sociodemographics

A total of 1246 respondents’ data were recorded through face-to-face interviews using conventional PPDC tools, whereas 1251 respondents’ records were observed in the electronic database. During data cleaning, we found that 5 of the records submitted through EDC were found empty. We removed the counter of 5 records from EDC because their records could not be depicted in the paper record files using the same IDs.

A total of 12 interviewers, 4 male and 8 female, aged 21 to 32 years, participated in the study. Among these, 4 were nurses, 3 were information technologists, 1 was a person with a background in social science, and the rest of the data collectors had no specific professional background. Regarding their educational status, the majority (8/12) had completed high school and vocational school certificate or diploma. Their experience as a field worker ranged from 2 months to 7 years with a mean of 2.6 years. Moreover, 4 of the interviewers had experience of using smartphones as a personal phone. None of the interviewers had previous experience in using a smartphone or tablet computers as a data collection tool.

### Unexpected Events During the Study

Although we started our survey with 6 pairs of data collector groups, unfortunately, one of the team terminated the study after 2 weeks of the survey period. The interruption occurred because of personal conflicts unrelated to the survey in the community. The conflict caused the termination of data collection in that area as the situation could not be resolved during the survey period. Therefore, we completed the survey with the remaining 5 groups.

### Study Findings and Outcome Data

#### Errors per Questionnaire

From the complete 1246 submitted questionnaires in both PPDC and EDC tools, 522 (41.9%) of the PPDC questionnaires had 1 or more errors. From this, the majority with 175 (33.5%) of the questionnaires that had only 1 error followed by 112 (21.4%) questionnaires with 2 errors and 55 (10.5%) with 3 errors.

**Figure 2:**
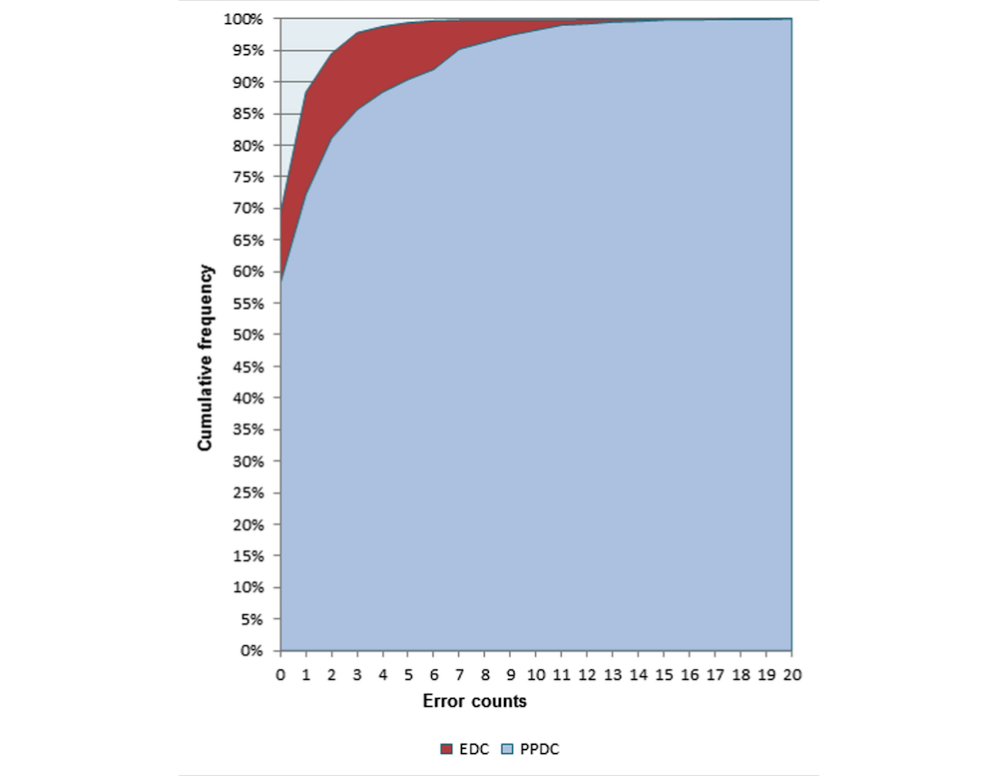
Frequency of error comparison among the electronic data capture (EDC) tools using tablet computer and paper and pen data capture (PPDC) tools during a survey in the demographic survey site in 2016, Dabat, northwest Ethiopia.

At EDC, 385 (30.9%) of the questionnaires had one or more errors. Thus, the majority of 241 (62.5%) had only 1 error followed by 76 (19.7%) questionnaires with 2 errors and 41 (10%) with 3 errors (Figure 2 and Multimedia Appendix 1).

#### Error Rate

The overall error rate, computed from the total error count over the total number of asked items, was 1.14%, from which 0.73% were missing and 0.4% of the rates were inaccuracy errors. The PPDC error rate was 1.67% (missing 0.92% and inaccuracy 0.75%), whereas the EDC error rate was 0.6%, of which 0.54% were missing and 0.064% were inaccuracy errors (Table 1).

#### Error Rate Over Time/Learning Effect

**Figure 3:**
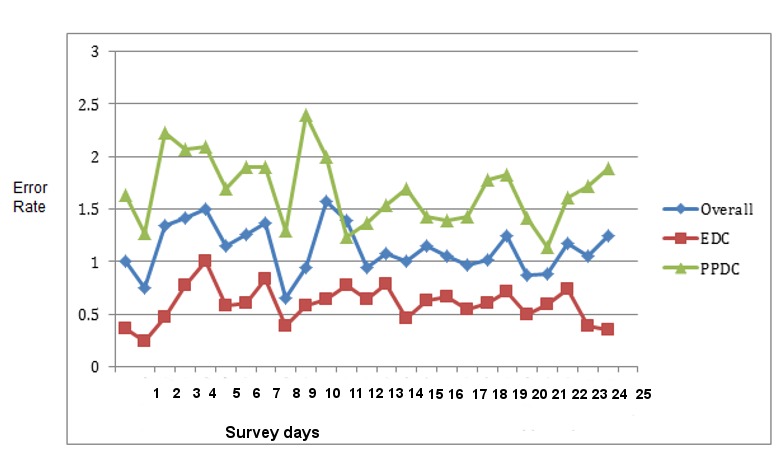
Mean values of the overall error rates trend of electronics data capture tool (EDC) using tablet and paper and pen data capture (PPDC) tools used during the survey in the demographic survey site in 2016, Dabat, northwest Ethiopia.

Though Figure 3 shows no smooth pattern of mean error rate increasing or decreasing over the study time, there is a visible difference observed between the 2 tools regarding the error rate. EDC has a constant error rate ranging below 1%, whereas the overall and PPDC error rates swing between 1% and 1.5%, respectively. There were random peaks of the error rates at different points of time in the study period; the overall error rate does not show a constant trend of decrease or increase over time (Figure 3). The statistical trend analysis also showed that trends are not detected at the *P*<.005 level.

**Table 1:** Error rate by types of errors and the tools used during a survey in a demographic survey site in 2016, in Dabat, northwest Ethiopia.

Error type	Error type		
	Overall asked items (N=221,106), n (%)	Paper-asked items (N=110,691), n (%)	Tablet-asked items (N=110,415^a^), n (%)
Missing	1620 (0.73)	1020 (0.92)	600 (0.54)
Inaccuracy	901 (0.40)	830 (0.75)	71 (0.10)
All errors	2521 (1.14)	1850 (1.67)	671 (0.60)

^a^The item difference results from the extra asked items in the paper questionnaire because of asking items that have to be skipped accordingly. This applies to other tables, too*.*

**Table 2:** Error by item type and the tools used during a survey in a demographic survey site in 2016, in Dabat, northwest Ethiopia.

Item types	Paper		Electronics	
	Total asked items (N=110,691), n	Number of errors (n=1850), n (%)	Total asked items (N=110,415), n	Number of errors (n=671), n (%)
Single select	46,660	466 (0.99)	46,427	145 (0.31)
Single select tabular	19,062	84 (0.44)	19,062	42 (0.22)
Multiple selects	24,303	877 (3.60)	24,302	226 (0.92)
Numbers and dates	18,022	343 (1.90)	17,980	202 (1.12)
Free text	2644	80 (3.02)	2644	56 (2.11)

#### Frequency of Errors per Item Type

Nearly half (47.4%) of the errors in PPDC were found in questions which had multiple option answers followed by questions with single choice answers (25.2%). At EDC, two-thirds of the errors were shared by multiple options, number and date type question with rates of 33.6% and 30.1%, respectively.

In the paper questionnaire, questions that had multiple answer options had a relatively higher rate of errors (3.65%), followed by free text and the number and date answers with 3% and 1.9%, respectively. In EDC, questions with free-text options had a relatively higher error rate with 2.1%, followed by numbers and date options answers (Table 2).

### Regression Results

In each estimated model, we found that the chance for a higher number of errors in the paper version (in comparison with the tablet version) increases with the length of the overall questionnaire. For each additional question in the interview, the chances of more errors by using the paper tool were multiplied by 1.015. We have obtained similar findings for the different subsets of the questionnaire, where we found an increase in the chances of additional errors in the paper version in each type of the questions. The increase is minimal for single-item questions. The increase is strongest in the time and date questions, with an odds ratio of 1.084 per additional question. None of the 95% CIs include an estimate of 0 and an odds ratio of 1, respectively (Table 3). Hence, we can see that each variable measuring a number of items has a significant effect on the comparative error rate.

### System Usability Scale Test

**Figure 4:**
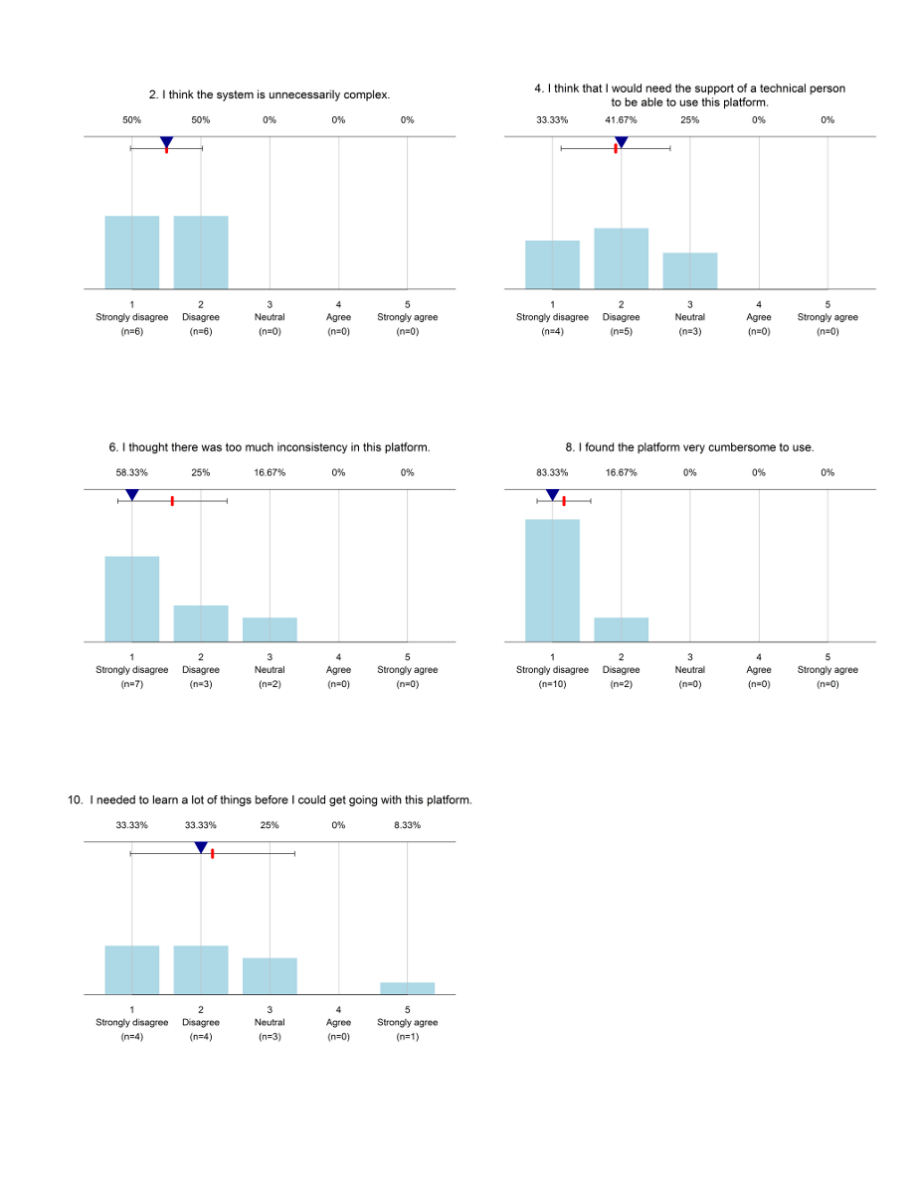
Usability response for negatively articulated questions in the System Usability Scale (SUS) used during a survey in the demographic survey site in 2016, Dabat, northwest Ethiopia.

**Figure 5:**
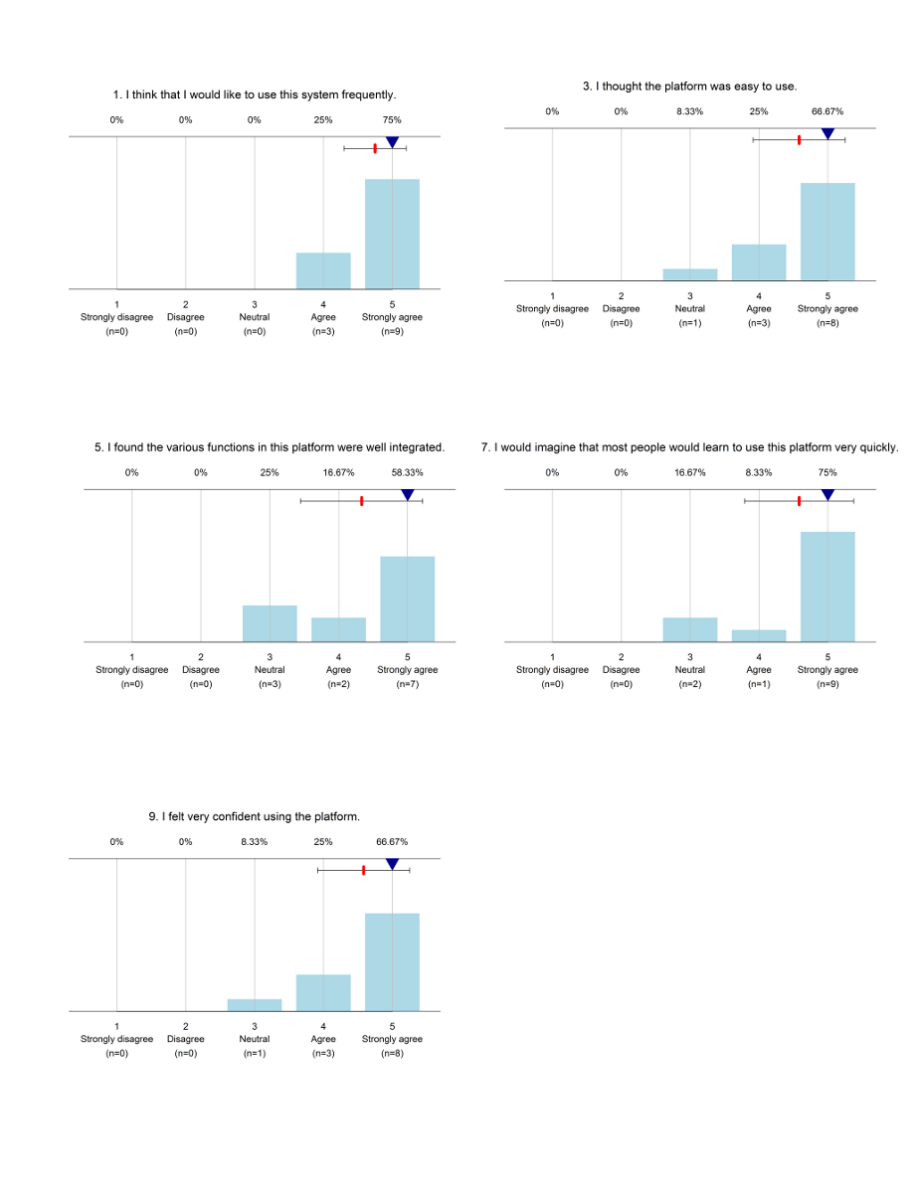
Usability questionnaire response for positively articulated questions in the System Usability Scale (SUS) used during a survey in a demographic survey site in 2016, Dabat, northwest Ethiopia.

The usability of the system among the data collectors was measured based on SUS. The global scores can range between 0 and 100, with 100 reflecting the highest usability. The analysis yielded individual SUS scores between 67.5 and 100, and a total average score of 85.6. The graph for each SUS question depicted that majority of the data collectors’ responses categorized from “agree” to “strongly agree” for positively articulated questions. Similarly, “strongly disagree” to “disagree” for the negatively articulated questions (Figures 4 and 5).

### Linked Qualitative Interview Themes With the International Organization for Standardization 9241—110 Dialogue Principles

As described in the Methods section, the qualitative interview responses of the system users’ perception were transcribed, thematized, and linked to one of the 6 ISO 9241-110 dialogue principles (suitability for the task, suitability of learning, controllability, self-descriptiveness, conformity with user expectation, and error tolerance). Users reported their perception by comparing the advantage of the EDC tool with respect to the disadvantage of paper-based questionnaire and vice versa.

According to the users’ perception, EDC tools increased the efficiency of the data collection process through faster data collection time, the possibility of instant data submission to the research center server, and real-time work progress. Users also perceived that EDC reduces potential data quality errors with its automatic data validity checks and skip errors rules. Furthermore, the EDC device was portable, lighter, and less bulky than paper questionnaires; users claim that exposure to EDC technology improved the skills of data collectors for information technology use in the survey. On the other hand, users also perceived the following about paper questionnaire: (1) they are prone to readability errors during correction/editing of respondent answers, (2) they can become out of stock while in the remote survey area, (3) paper is heavy and easily wasted during improper handling or storage, and (4) they can incur higher duplication and operational costs for larger surveys. The perceived benefits of EDC combined with limitations of paper questionnaire was interpreted as—EDC had good suitability of task, ET, controllability, and suitability of learning for the above listed tasks.

Users also perceived that EDC possesses a high risk of losing data because of intentional and unintentional technical mistakes from the user or device failures. The fact that EDC needed sustainable electricity and internet infrastructures for charging the tablet computers and for facilitating instant data transfers was also considered as a treat to work in underserved deep rural towns. The data collectors raised a concern that the deep rural towns usually have neither of them. However, users’ familiarity with the paper questionnaire, easiness of paper to correct questionnaire related mistakes, lesser risk of losing data for technical reasons, and less dependency on electricity or internet infrastructures for daily operations were referred to as the advantages of the PPDC tool. Thus, the disadvantages of EDC coupled with perceived benefits of PPDC characterize EDC to be less conformable with user expectation, controllability, ET, and suitability for the above tasks.

The views of the interviewee were also further mapped based on aspects explored during the interview, which include, efficiency/speed, data recording and entry processes, data management/operation, logistics and operation, concern, learning, infrastructure, reported and solved challenges, satisfaction, and confidence and future improvement (Table 4).

**Table 3:** Mixed model effect, ordinal logistic regression for the data collected by electronic data capture (EDC) and paper and pen data capture (PPDC) tools used during a survey in the demographic survey site in 2016, in Dabat, northwest Ethiopia.

Covariate	Regression coefficient b_1	Exponential(b_1) multiplicative change in chances	SD (95% CI)
Total number of items	0.015	1.015	0.0035 (0.008-0.021)
Number of single items	0.029	1.030	0.0074 (0.014-0.044)
Number of multiple items	0.042	1.043	0.0094 (0.024-0.060)
Number of single table items	0.023	1.023	0.011 (0.001-0.045)
Number of time and date items	0.081	1.084	0.017 (0.048-0.114)

**Table 4:** Data recorders’ perceptions and its isometric dialogue mapping for data capture tools used during a demographic survey site in 2016, in Dabat, northwest Ethiopia.

Aspect explored and data collectors’ or users’ perceptions		EDC^a^ dialogue mapping (+/−)^b^
**Efficiency or speed**		
	Paper questionnaire takes less time than the electronic questionnaire, more data can be collected in less time, as it is more familiar and easier to write on.	ST^c^−
	If the device is free of internal software or hardware errors, working with tablet computer is relatively faster than paper, and this can save overall working time.	ST+
**Data recording and entry processes**		
	In the paper questionnaire, deletion or editing respondent response while editing written mistakes or incorrect response can cause readability errors. In tablet computers, we can easily delete or edit responses without causing readability errors.	ET^d^+ and ST+
	With the paper questionnaire, we may forget the skip question pattern and ask the follow-up questions, but in the case of using tablet device, there is an automatic skip pattern function to lead the questioning order	C^e^ or SDS^f^+
	In EDC, the data can reach the concerned bodies faster by jumping the double data entry clerk and other data management processes compared with the paper-based system.	ST+
	Data collected with EDC has fewer errors than paper questionnaires as the electronic form has an error controlling validity mechanism.	ET+
	Mistakes in questionnaire items were possible to erase or correct in EDC form, whereas in paper form, it is possible to edit or erase the items manually.	C− or ST+/−
	A simple technical error in the table computer can affect the whole work. For example; in the case of unknown or unintentional error in the device, there is a risk of losing the whole data, and reinterviewing the respondent is a must.	C− and ET−
	Writing on tablet virtual keyboard, moving the cursor, and editing text fields were a challenge and took a longer time. On paper questionnaire, the interviewer can easily write or circle the chosen item and pass to the next question.	C− and ST−
	ODK^g^ collect app menus, such as get a blank form, fill the blank form, edit saved form, and send finalized form, that were easily understandable.	ST+ and SL+
**Data management or operation**		
	Well-documented paper questionnaires or files can stay longer and are not prone to file corruption or deletion errors as in the case of electronic data collection system.	ST− and ET
	In the paper form, if mistakes were found a while after completing the interview, it can be corrected before submitting the questionnaire to the supervisor; however, in EDC, we do not have a chance to correct later as the data are sent instantly to the server while we are in the study field.	ST+
	There is a high possibility of data loss in paper questionnaires because sheets of paper suffer from wear and tear during transit and storage.	ST+
	Actual work progress of the data collectors was better monitored with daily and instant submission of the completed questionnaire through EDC.	ST+
**Logistics and operation**		
	The paper questionnaire runs out while we are in the field and sometimes it takes longer to reach, which is not the case in EDC.	ST+
	The tablet computer is small and easy to handle, whereas the paper questionnaire pack was heavy and bulky. Carrying paper packs house to house for longer distance was difficult and had physical stress.	ST+
Security concern: Data collectors feel insecure to work in rural areas alone with this expensive device. Furthermore, the device or the data recorders might be forcefully stolen or may be attacked in case of theft attempt.		ST−
Learning opportunity: Data collector’s skill of tablet computer utilization was increasing day to day as familiarity with the system was growing.		SL^h^+
**Infrastructure**		
	The battery capacity lasts a maximum of a one and a half days; during the study period, overnight charging of the tablet computer in or around the research town was possible.	ST+
	Data collectors appreciated the role of paper-based system when their work was interrupted because their tablet battery was low or switched off or when the mobile data connection was interrupted.	ET−
	Internet collection was adequate to send the data once a day during the study period.	ST+
	Sometimes in deep rural areas, electricity may not be available to charge the battery or adequate enough for the internet network coverage to be available.	ST−
**Reported and solved challenges**		
	Downloading a new form at the beginning of the study or during form updating was challenging.	SDS and C−
	Deleting partially filled data was challenging.	C −
	Some questions in EDC form were refusing to accept the decimal number during data collection in the first 2 days.	CUE^i^−
	Unintentional and accidental touching of some settings in the table computer changed the screen resolution settings, the date and time, and interrupted communication with the server.	C−
	Mobile internet data balance was exhausted while the data collectors were in the remote area where charging the balance was not possible.	ST−
Satisfaction and confidence: The data collectors have better satisfaction with EDC and have confidence to work using this device in future surveys.		ST+
**Future improvement**		
	The questionnaire details such as point, number, and legal value, should be well refined in EDC before implementation.	CUE−
	As frequent phone consultation for technical help may not be possible in case of network problems, strong training is needed to troubleshoot simple errors by EDC users without technical team consultation.	CUE−UE

^a^EDC: electronic data capture.

^b^+ corresponds to more and − corresponds to less.

^c^ST: suitability of task.

^d^ET: error tolerance.

^e^C: controllability.

^f^SDS: self-descriptiveness.

^g^ODK: open data kit.

^h^SL: suitability for learning.

^i^CUE: conformity with user expectation.

### Unexpected Observations

Most of the household face-to-face interviews went smoothly except for a few incidences such as loss of internet connectivity, unexpected electric power interruption in the area, and software bugs. Recording geographic coordinates were part of the EDC questionnaire and was a required input. Detecting the global positioning system (GPS) signals was sometimes a challenge for our 3 survey sites. This required option had created a substantial delay in the data collection process for 3 groups, as further questions could not be asked without detecting the GPS signals. As a quick solution, we removed the required option from the app to resume data collection processes.

## Discussion

### Answer to the Study Questions

This study aimed to evaluate and compare the magnitude of data quality errors in the data collected with paper and pen as well as EDC technologies. In addition, the usability of the tablet computer to capture survey respondent data was analyzed from the perspective of data collectors. The answer for these study questions are arranged in magnitude of errors, learning effect, quantitative SUS evaluation, and isometric qualitative evaluation for system usability.

#### Magnitude of Data Quality Errors

In DHS, data collection systems are expected to be sustainable, easy to use, provide timely data, be reliable, and maintain data quality. Our evaluation study demonstrated that a smart device data collection system using the ODK platform outperformed pen-and-paper systems across each data quality parameter for DHS in Ethiopia. Data collected using tablet computers were more likely to have fewer errors compared with the conventional paper questionnaire. Nearly half of the PPDC and one-third of EDC questionnaires exhibited one or more errors in the respondent answers.

The data collection mode features can affect response quality. Face-to-face or personal-visit surveys have a high degree of interaction with the respondent irrespective of the technology use. Interviewers can also support in clarification, probing, and encourage the respondents to provide complete and accurate responses [33]. The conceptual reasons why errors might be less likely to occur when using technology, particularly when human-technology interaction is inevitable, need further explanation. Data quality in general possesses completeness, plausibility, and validity dimensions. Addition of technological support for face-to-face interview mode targeted on the improvement of the completeness and plausibility data quality dimensions. EDC timely avoids completeness and plausibility errors through automated routing in complex questionnaires and detecting errors using range and consistency checks and warning interviewers of invalid entries. Though improving completeness and plausibility contribute to validity dimension, further research is needed to assess the validity dimension. In addition to improving the design of the instrument with principles of human-computer interaction and user-centered design, appropriate training on error recovery procedures to avoid potentially deleterious consequences (eg, accidental deletion of files) is important [33,34].

It is a very challenging task to find a theoretical base for the superiority of technological support. We can think of the Charles Friedman’s fundamental theorem for biomedical informatics. It states that “a person working in partnership with an information resource is ‘better’ than that same person unassisted.” The literal translation to us is “a field-based interviewer who use electronics data collection system for the survey will make fewer data quality errors than the interview administrator without the electronic system.” Though full consensus has not been reached on interpreting the theorem, Friedman’s theorem is the most arguable applied theorem in the informatics community [35].

Computing error rate, which considers the total number of questions, is important in case of bulky questionnaires such as ours. For example, the error rates for PPDC and EDC tools were 1.67% and 0.6%, respectively. This shows that most of the submitted questionnaires in both tools had very few error counts compared with the total number of asked items in a questionnaire. The majority, that is, 85% of EDC and 55% of PPDC had less than 3 errors from 38 questions in the average questionnaire.

The low rate might be from 2 possible speculated effects; the first is “being observed or supervised effectˮ on the data collectors and the second might be “inter-tool/interaction among the data collectors.ˮ The data collectors feel that their performance can be watched indirectly and making mistakes can have an unintended consequence in their job. Extra care might have been taken by the data collectors to avoid potential mistakes while working. The intertool interaction between the paired data collectors might affect the performance of one of the data collector over the other pair. Though we have strictly instructed the interviewers to avoid any side communication during the interview, they might communicate indirectly in the sense of helping each other during technical challenges. This unwanted communication might neutralize the data quality of impact of a tool over the other. Further analysis of paired datasets in the same team supported this claim, where 3 of the 6 paired groups have similar magnitudes of errors between the paired individuals. The effects were unavoidable because of our study design nature where the paired data collectors must conduct the interview.

Missing and inaccuracy errors were analyzed separately, and the percentages indicate both types of errors were more prevalent in the paper-based system. Missing errors are more prevalent than inaccuracy errors in both tools. However, inaccuracy errors in electronic-based data are quite low, 10.5% (71/671), compared with a paper questionnaire, 81.37% (830/1020). This might be because electronic form had partial programmed checking mechanisms such as skip pattern. On the other hand, from the total errors count (671) observed in electronic data, 600 (89.5%) errors were because of missing items. This accounts for 37% of the total errors in both tools. Considering previous experience and the nature of electronic device for minimal tolerance of missing values, the magnitude seems quite high. This is expected because of the study design nature. In a research design such as ours where 2 data collectors sit next to each other, they start and finish the study at approximately the same time, implementing all possible validation rules and complicate the study design. For example, in case the person with the tablet is leading the interview and the person with the paper follows or vice versa, one of them does not have to wait for the other for any mismatch reasons. Implementing strict validation rules will create interdependency or confusion among them. This has been observed in our pretest phase, and hence, we have deliberately omitted some of the validation rules from the EDC.

The possession of more errors in paper compared with EDC were also supported by our qualitative interview data. The majority of the data collectors perceived EDC had minimized the potential errors through inbuilt error controlling validity tool and systematic routing with skip logic.

#### Learning Effect

A learning curve usually describes technological progress (measured generally in terms of decreasing error rate for EDC) as a function of accumulating experience with EDC over time. It assumes that a technology’s performance improves with experience when the technology accumulates. In this study, a single data collector had a cumulative 2 weeks exposure in the 4-week study period. In this short time exposure, performing early assessment is open to criticism as the users are not fully proficient. Late assessment runs the risk that the data collectors will draw their own conclusions about the pros and cons of the new technology. In our case, both the statistical tests and graphical illustrations did not depict any meaningful time pattern on the error rate through the study period. This might be because the interviewers were exposed equally to each tool at random order to avoid any unnecessary learning effect on the quality of the data, which can be sourced from uneven prolonged exposure of the user to one of the tools.

#### System Usability Scale and Isometric Usability

SUS scores and impression of the data collectors from the interview showed high usability and satisfaction in using EDC for data collection with a usability score of 85.4. Data collectors stated that the system is easy to carry, they were able to directly submit the data to the server, and let them be familiar with the information technology; this encourages the implementation of such systems in resource-limiting settings.

Analysis of the qualitative interview based on the isometric dialog principles for usability identified perceived strengths and areas of improvements to implement the electronic data collection system. For example, the system had better ET because of its error controlling mechanism and less ET for its high risk to lose the data.

The qualitative responses were triangulated with isometric dialogue principles. The conceptual focus of these dialogues is basically about the hardware and software capability system for the given task. However, we tried to force some of the dialogue principles to accommodate environmental and social contexts that are of particular importance to the research sites settings. For example, responses such as, “we had a constant supply of electricity and internet,” “I work relatively faster when I use a tablet computer,” and “When I use a tablet, I will not worry about finishing the questionnaire” were categorized under suitability for task dialogue principle. The responses are neither software interface issues nor hardware issues, rather, they are social, economic, and infrastructural setting context to facilitate the data collectors’ tasks. This may imply that there could be room to extend the dialogue principles on the importance of local context issues where the system evaluation takes place. Though mapping the qualitative interview cannot explicitly show all the dialogue principles into the level of equal share, relevant information can be drawn even with its limitations. For the future, a qualitative research design where the interview questions intended to include all the 6 dialogue principles can yield a better understanding of the system usability.

Tablet computers are valuable items in resource-limited settings; thus, the device can be stolen or data collectors may be targeted when conducting household surveys in insecure areas. The potential incidence of theft and subsequent physical attacks or fear of losing the device were frequently mentioned concerns by the data collectors. Similar concerns were raised by other researchers in South Africa; such types of insecure feelings may create unintended resistance from the field interviewer. If the system planned for scaled-up implementation in the routine work, addressing their concern is an important step for successful integration of the system in the area.

### Strengths and Weaknesses of the Study

This study adopted an existing open source platform to develop the electronic questionnaire and data submission and storage configuration. This showed we could easily adopt the available resources and save the human and material resources to develop the new system from scratch.

The research design in this study is a crossover, randomized trial, which helps to eliminate unnecessary learning trends and shows the real effect of the devices accordingly.

The other possible strength of this study is the fact that the survey showed the usability of the system using the quantitative (SUS) and the qualitative interview supported by isometric dialogues principles.

Pairing data collectors for a single interview may affect the interdata collectors’ performance or tools may affect each other, hence lowering the magnitude of actual errors. Due to the inability to implement all the functions of the electronic error controlling mechanism, such as required items for all, value range determination in EDC might have contributed to some preventable errors that existed in EDC.

Though interviewer effects appear not to affect most survey items, literatures showed that interviewer characteristics effects such as race, ethnicity, and gender can nonetheless occur in all interviewer-administered survey modes and can affect survey findings. If these effects are replicated because of use of small number of interviews, it might result in a bias. For our study, data collectors were randomly exposed to either an electronic device or a paper questionnaire. Though the existence of bias is unavoidable, we believe the effect is similar in both tools [36].

### Results in Relation to Other Studies

The overall error rate in this study, 1.67% for PPDC tool and 0.6% for EDC is comparable with a study in Kenya that had 1% and 0.1% missing rate in paper and electronics, respectively [11]. In addition, a study with a similar design in India revealed 2% PPDC and 1.99% EDC error rates [29]. However, this magnitude is lower compared with other similar studies (7% and 1%, with no omission in EDC) [9], 3.6% PPDC and 5.2% in EDC [37]. The difference might be an item selection mechanism: the above studies calculated their error rate from selected items in a given questionnaire, whereas this study considered all items in the questionnaire, which is highly likely to inflate nonerror denominators for such types of typical huge number of asked items (over 220,000). Furthermore, random assignation of data captures tools for data collectors, whereas the studies compare data collected from 2 different points in time by various data collectors and nonrandomized methods. Nonrandomized studies might possess inflated error influenced by nonhomogeneous factors distributions among data collectors and other characteristics of the study.

Many EDC studies that use the maximum potential of error control mechanisms such as required items for all, logical skip, or logical input values in EDC, reported 0 to less than 1% of missing responses [14,38]. In our study, because of the design nature mentioned earlier, we could not fully implement all the error-controlling mechanisms in EDC. Under possible circumstances, implementing the maximum potential of the error preventing mechanism in the software is mandatory.

Our study and other related findings revealed there is a statistically significant difference in the chance of having more errors in paper-based records than in electronic data records [7,9,11]. However, an equivalent research report from rural India claims that there was no statistical difference in error magnitude between the 2 tools, where EDC is as accurate as PPDC [29]. In our study, the chance of having more errors increased as the number of questions asked increased. Our study was unique in depicting this finding, because of the nature of the survey. In this survey, all the household respondents were not necessarily given the same type of questionnaires and number of questions. The number of questions and the type of the questionnaire depends on the type of public health cases that existed in the household members interviewed by the data collectors. The implication might be the length of the questionnaire, and this might be an important factor to be considered in the decision of choosing the type of data capture tools during design. Bulky questionnaires might be more suitable for electronic records than a paper-based system.

The SUS score value 85.4 and the qualitative interview isometric dialogue mapping shows the system users, in our case data collectors, use the system comfortably. These findings were shared by other EDC users’ impressions in related researchers [11,26,29,37,38]. At ask-technology fit study claims that users understand their system as tools helping or hindering them from performing their tasks. Users react positively to system features that appreciate their mission-related demands [39].

### Meaning and Generalizability of the Study

Our study findings are in line with similar studies that used the ODK app platform to develop the electronic form and the data management settings and implemented in rural and urban community household surveys, though there is a methodological difference. The quantitative and qualitative analysis depicted that EDC is a usable and preferable data capture tool for the field-based survey.

We believe the study might be generalized to rural community setting research sites, with limited internet and electricity access.

### Unanswered and New Questions

Working in a pair might put an “intersupervisorˮ effect on the data collector’s performance. In a further study, we can assign a data collector independently to work with EDC and avoid the intersupervisor effect. Moreover, measuring the learning effect of the technology after longer exposure with the system and using appropriate statistical techniques might give a different result.

Moreover, research considering organizational readiness evaluation, working culture alteration, and its outcome measurement in using this system in resource-limited settings are tasks on the table. Further research is also necessary to validate qualitative-based isometric usability questions in different linguistic and communication culture settings.

### Conclusions

The objectivist approaches of this study conclude that data collected by using an electronic-based data collection system had a significantly better quality compared with a paper-pen data collection system explained by fewer errors. Implementation of electronic data collection tools such as the ones tested in this comparative trial were found to be usable by data collectors in the rural resource-limited settings. Implementation of a full error-controlling function exhaustively, setting a standby technical and monitoring team, and assuring security concerns on the device will contribute to better implementation of the electronic data collection system in the resource limited-settings. Stakeholders of the health information system particularly in a demographic and surveillance site can adapt and use the existing open source mobile device platforms in their routine data collection and management practices.

## Appendix

Multimedia Appendix 1 Supplementary table for the frequency of error in electronic data capture and the paper data capture tools.

Multimedia Appendix 2 Sample picture of data collection setting where the 2 data collectors with tablet computer and paper questionnaire conduct the survey simultaneously.

Multimedia Appendix 3 A semistructured questionnaire used to interview the system users.

## Abbreviations

C: controllability
CUE: conformity with user expectation
DHS: Demographic and Health Surveys
EDC: electronic data capture
ET: error tolerance
GPS: global positioning system
HDSS: Health and Demographic Surveillance System
ID: identification
ISO: The International Organization for Standardization
mHealth: mobile health
ODK: open data kit
PPDC: paper and pen data capture
SDS: self-descriptiveness
SL: suitability for learning
ST: suitability of task
SUS: System Usability Scale
