# Supplementary material for: Evaluation of Electronic and Paper-Pen Data Capturing Tools for Data Quality in a Public Health Survey in a Health and Demographic Surveillance Site, Ethiopia: Randomized Controlled Crossover Health Care Information Technology Evaluation
Source: JMIR Mhealth Uhealth. 2019 Feb 11;7(2):e10995. doi: 10.2196/10995 (PMC6388101; doi:10.2196/10995)
Supplement: Multimedia Appendix 1 [file mhealth_v7i2e10995_app1.pdf]

Supplementary information for frequency of error among EDC using the tablet computer and paper and pen data capture (PPDC) tools during a survey in the Demographic survey site in 2016, Dabat Northwest Ethiopia

Error count	Paper and pen data capture (PPDC) tool			Electronics data capture (EDC) tool		
	Frequency	Percent	Cumulative	Frequency	Percent	Cumulative
0	724	58.1	58.1	861	69.1	69.1
1	175	14.0	72.2	241	19.3	88.4
2	112	9.0	81.1	76	6.1	94.5
3	55	4.4	85.6	41	3.3	97.8
4	35	2.8	88.4	12	1.0	98.8
5	26	2.1	90.4	7	0.6	99.4
6	19	1.5	92.0	4	0.3	99.7
7	40	3.2	95.2	1	0.1	99.8
8	14	1.1	96.3	0	0.0	99.8
9	14	1.1	97.4	0	0.0	99.8
10	9	0.7	98.2	0	0.0	99.8
11	11	0.9	99.0	1	0.1	99.8
12	2	0.2	99.2	1	0.1	99.9
13	4	0.3	99.5	0	0.0	99.9
14	1	0.1	99.6	0	0.0	99.9
15	2	0.2	99.8	0	0.0	99.9
16	1	0.1	99.8	0	0.0	99.9
17	1	0.1	99.9	0	0.0	99.9
18	0	0.0	99.9	1	0.1	100.0
19	0	0.0	99.9	0	0.0	100.0
20	1	0.1	100.0	0	0.0	100.0
Total 1246		100.0	Total 1246		100.0	
