# Supplementary figures and images for: Evaluation of Electronic and Paper-Pen Data Capturing Tools for Data Quality in a Public Health Survey in a Health and Demographic Surveillance Site, Ethiopia: Randomized Controlled Crossover Health Care Information Technology Evaluation
Source: JMIR Mhealth Uhealth. 2019 Feb 11;7(2):e10995. doi: 10.2196/10995 (PMC6388101; doi:10.2196/10995)

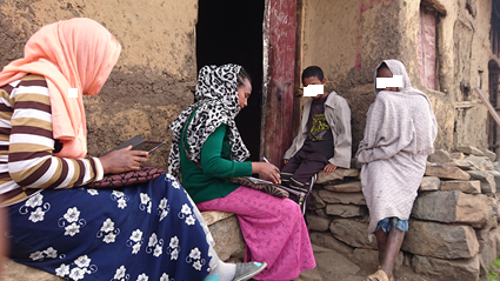

Supplement: Multimedia Appendix 2 [file mhealth_v7i2e10995_app2.png]
