# Supplementary material for: Evaluation of Electronic and Paper-Pen Data Capturing Tools for Data Quality in a Public Health Survey in a Health and Demographic Surveillance Site, Ethiopia: Randomized Controlled Crossover Health Care Information Technology Evaluation
Source: JMIR Mhealth Uhealth. 2019 Feb 11;7(2):e10995. doi: 10.2196/10995 (PMC6388101; doi:10.2196/10995)
Supplement: Multimedia Appendix 3 [file mhealth_v7i2e10995_app3.pdf]

A semi-structured questionnaire used to interview the system users

i.	Age	Response
ii.	Sex	
iii.	Educational status	
iv.	Profession	
v.	How long is your experience as data collector?	
vi.	Do you have experience with smart phones?	
vii.	Have you any experience with EDC?	
1.	From your previous experience as a data collector how do you explain the merit and the challenge for paper based data collection processes?	
2.	How do you feel the first time you start to use EDC as a means of data collection?	

3.	From your experience of using EDC as data collection tool in this study period, how to you describe the benefit or the limitations of using EDC as data collection? What are the challenged you have faced in using the system?	
4.	Do you think the current infrastructure in your site (electricity and network) is good enough to use this electronic data collection system?	
5.	How many days on average you got data collection interruption because of either Internet disconnection, technical issue?	
6.	Which part of the system functioning (getting new form, filling blank form , editing and saving form and sending final form) do you understand it easily and which part was difficult during your day to day data collection with EDC? And how do you solve the difficulties?	
7.	To what extent are you satisfied with the system? what are the functions EDC of the data collection process which makes you satisfied compared to compared based medical records	

8.	With your current performance are you able to collect data with only EDC without fear of Deletion of forms, or other technical errors committed by you?	
9.	How do you think EDC is appropriate to support the work of your current organization? Why?	
10.	How was the response of the interviewee when you use EDC as a means of data collection tool compared to the paper based tools?	
11.	What kinds of unexpected events has happened during data collection using EDC how do you solve it	
12.	Are you interested in using the electronic data collection for the future? Why	
13.	What kinds of future improvement you would like to see in this EDC?	
14.	Do you have any final comment?	

Thank you for your cooperation for this interview
